# Dynamic connectivity patterns of resting-state brain functional networks in healthy individuals after acute alcohol intake

**DOI:** 10.3389/fnins.2022.974778

**Published:** 2022-09-20

**Authors:** Gengbiao Zhang, Ni Li, Hongkun Liu, Hongyi Zheng, Wenbin Zheng

**Affiliations:** ^1^Department of Radiology, The Second Affiliated Hospital, Shantou University Medical College, Shantou, China; ^2^The Family Medicine Branch, Department of Radiology, The First Affiliated Hospital, Shantou University Medical College, Shantou, China

**Keywords:** alcohol, functional magnetic resonance imaging, dynamic connectivity, functional connectivity, brain functional network, healthy volunteers

## Abstract

**Aims:**

Currently, there are only a few studies concerning brain functional alterations after acute alcohol exposure, and the majority of existing studies attach more importance to the spatial properties of brain function without considering the temporal properties. The current study adopted sliding window to investigate the resting-state brain networks in healthy volunteers after acute alcohol intake and to explore the dynamic changes in network connectivity.

**Materials and methods:**

Twenty healthy volunteers were enrolled in this study. Blood-oxygen-level-dependent (BOLD) data prior to drinking were obtained as control, while that 0.5 and 1 h after drinking were obtained as the experimental group. Reoccurring functional connectivity patterns (states) were determined following group independent component analysis (ICA), sliding window analysis and k-means clustering. Between-group comparisons were performed with respect to the functional connectivity states fractional windows, mean dwell time, and the number of transitions.

**Results:**

Three optimal functional connectivity states were identified. The fractional windows and mean dwell time of 0.5 h group and 1 h group increased in state 3, while the fraction window and mean dwell time of 1 h group decreased in state 1. State 1 is characterized by strong inter-network connections between basal ganglia network (BGN) and sensorimotor network (SMN), BGN and cognitive executive network (CEN), and default mode network (DMN) and visual network (VN). However, state 3 is distinguished by relatively weak intra-network connections in SMN, VN, CEN, and DMN. State 3 was thought to be a characteristic connectivity pattern of the drunk brain. State 1 was believed to represent the brain’s main connection pattern when awake. Such dynamic changes in brain network connectivity were consistent with participants’ subjective feelings after drinking.

**Conclusion:**

The current study reveals the dynamic change in resting-state brain functional network connectivity before and after acute alcohol intake. It was discovered that there might be relatively independent characteristic functional network connection patterns under intoxication, and the corresponding patterns characterize the clinical manifestations of volunteers. As a valuable imaging biomarker, dynamic functional network connectivity (dFNC) offers a new approach and basis for further explorations on brain network alterations after alcohol consumption and the alcohol-related mechanisms for neurological damage.

## Introduction

Alcohol has a deep and enduring history of association with human culture and it is widely used in healthcare, industries, catering, social occasions, etc. (Sudhinaraset et al., 2016; Khaderi, 2019). In recent years, there has been a worldwide increase in the demand for alcoholic beverages (Geneva: World Health Organization, 2018). People who drink alcohol may experience exhilaration, but drinking is associated with several health and safety risks that may outweigh any potential benefits. Topiwala et al. (2017) and Immonen et al. (2020) pointed out that even moderate drinking can cause damage to the brain. Consumption of alcohol over a prolonged period can also result in chronic damage to the brain, which can lead to impaired brain function. Currently, most alcohol and brain literature focus on the chronic effects alcohol has on the brain (Rogers et al., 2012; Mueller-Oehring et al., 2015), with only a few studies examining the acute effects of alcohol. However, acute alcohol consumption can also cause significant changes in brain function and behavior, which often lead to a series of risky behaviors, including drunk driving and violence (Norström and Pape, 2010; World Health Organization, 2018).

The primary emphasis in the majority of previous studies was placed on the spatial properties of brain function following acute alcohol exposure. By resetting brain function to a relatively stable state over an extended period, researchers were able to study the changes in brain regions with different spatial coordinates or sub-networks of the brain throughout the corresponding time. However, the most recent research by Hutchison et al. (2013) demonstrated that the brain is a complex dynamic system that constantly changes on a microscopic time scale to adapt to the environment. Therefore, relevant studies are limited to spatial properties without considering temporal properties. The dynamic functional connectivity (dFC) analysis with the sliding window approach considers the brain’s dynamic temporal changes (Allen et al., 2014), hence providing additional information not available with static functional connectivity analysis. As research on the temporal properties of brain function continues to advance, dynamic functional network connectivity (dFNC) analysis has been extensively used to study schizophrenia (Du et al., 2016), Parkinson’s disease (Diez-Cirarda et al., 2018; Fiorenzato et al., 2019), and autism spectrum disorders (Fu et al., 2019). The dFNC is also applied to alcohol use disorder patients. Gerchen et al. (2021) discovered that the temporospatial variability of dynamic PeaCoG (“peak connectivity on a gradient”) in the left dorsolateral prefrontal cortex is reduced in Alcohol use disorder and this reduction is associated with self-efficacy to abstain and duration of abstinence. The dFC has not yet been used in the study of brain functional networks during acute alcohol exposure. A previous study (Zheng et al., 2015), discovered that acute alcohol intake affected the functional network connectivity state of the brain, which might provide a neural basis for alcohol’s effects on behavioral performance. We hypothesized that there could be simultaneous temporal changes in corresponding brain networks and there may even be specific patterns of dynamic functional connectivity under acute alcohol exposure and that the corresponding patterns could help explain the underlying mechanism of the effect of alcohol on the brain network.

Our research group intends to use the sliding window approach to study the dynamic changes in the brain’s functional network connectivity state that occur in healthy volunteers following acute alcohol intake, using resting-state functional magnetic resonance imaging (rs-fMRI). This study may provide new insight into the mechanism of action of alcohol on the brain network, hence facilitating further research into the topic.

## Materials and methods

### Participants

Twenty healthy right-handed volunteers (11 males and 9 females) aged 23–31 years old (mean: 25.10 ± 2.27 years) were enrolled, and all had no history of alcohol abuse or psychiatric/neurologic diseases such as anxiety or depressive disorders. In order to ensure the reliability and security of the study, individuals with poor tolerance or allergies to alcohol were excluded. All volunteers accepted routine brain MRI scans and related physical examinations to rule out anatomical brain abnormalities and other systemic diseases. Female volunteers were required to participate in the non-menstrual period. Besides, the volunteers were requested to avoid consuming strong tea, coffee, spicy food, alcoholic food or beverages for 24 h and to refrain from eating for 6 h prior to the experiment. Moreover, the volunteers must undergo an alcohol breathalyzer test before the experiment to ensure they were free of alcohol.

All volunteers were informed of the amount of alcohol intake and contraindications for MRI and signed the Informed Consent. The Ethics Committee approved the study of the Second Affiliated Hospital of Shantou University Medical College.

### Procedure

Before alcohol intake, all the volunteers underwent thin-slice T1WI and blood oxygen level-dependent (BOLD) scans, with the data obtained as control. Then, they were instructed to consume Chinese Baijiu “Qinghua Fenjiu 20” (53% VOL, produced in Shanxi Province, China) at 0.65 g/kg alcohol dose together with some food (e.g., peanuts, beef jerky) within 10 min. Alcohol consumption was calculated based on weight with the following equation: alcohol consumption (milliliters) = the amount of alcohol intake (grams)/alcohol concentration (percentage) × 0.8 (ethanol attenuation). Subsequently, each volunteer underwent a BOLD scan and performed a self-assessment for dizziness, headache, nausea, vertigo, flushing, etc., 0.5 and 1 h after alcohol intake. Breath alcohol concentration (BrAC) was determined before BOLD using the Alco-Sensor III breathalyzer (ALCPRO, Knoxville, Tennessee) (Borges et al., 2004) and used to estimate blood alcohol concentration (BAC).

### Magnetic resonance imaging acquisition

The GE 3.0T MRI scanner with a standard 8-channel phased-array head coil was utilized for MRI scans. The participant’s head was immobilized with sponge pads to reduce movement artifacts and earplugs were used to lessen the impact of noise. After routine three-plane localizer scan, axial three-dimensional brain volume (Ax 3D-BRAVO) T1WI thin-slice scan using a gradient echo (GRE) sequence was conducted [slice thickness = 1.2 mm, slice gap = 0 mm, time of repetition (TR) = 7.8 ms, time of echo (TE) = 3 ms, field of view (FOV) = 240 mm × 240 mm, matrix = 256 × 256, flip angle = 15°, number of slices = 248]. The whole brain was scanned from the cranial vault to the foramen magnum, with the anterior-posterior line as the scanning baseline. Rs-fMRI data were obtained using a single-shot GRE echo-planar imaging (EPI) sequence, and BOLD data were obtained from the sites with identical 2D anatomical images. The scanning parameters were set as follows: TR = 2,000 ms, TE = 30 ms, slice thickness = 5 mm, slice gap = 0 mm, FOV = 240 mm × 240 mm, matrix = 64 × 64, flip angle = 90°, and number of slices = 25. After 7-min scanning, 5,250 original DICOM images were collected from 120-time points. All participants were awake and calm with their eyes closed during the scanning process.

### Image preprocessing

Structural and functional MRI data were preprocessed using the SPM8 and RESTplus V1.211 following steps. Initially, the original DICOM images were converted to NIFTI format. Data of the first 10 time points were discarded to eliminate the effect of magnetization inhomogeneity. Then, the functional images were slice-time corrected to ensure unified scan time of each layer in a scanning cycle and subsequently were head-motion corrected to correct image displacement and exclude data with head movement > 3.0 mm across the translational direction and > 3.0° across the rotational direction. Afterward, the functional images were normalized to the axial Montreal Neurological Institute (MNI) EPI template of the SPM software. The images were then spatially smoothed using a Gaussian kernel of 6-mm full-width at half maximum (FWHM). Head motion of all subjects was analyzed before and after acute alcohol intake and no statistical difference was found (Supplementary Table 1 provides details of the statistical results of head movement parameters).

### Group independent component analysis

The preprocessed data were decomposed into functional networks through group independent component analysis (GICA) as implemented in the GIFT v4.0a software.^1^ A higher-order GICA method utilized by Kiviniemi et al. (2009). was adopted to improve functional parcellation using the information maximization (Infomax) algorithm (Bell and Sejnowski, 1995). A total of 100 independent components (IC) were divided and compared to 100 iterative estimates *via* ICASSO method as implemented in the GIFT software to assess the reliability of the estimation by Infomax ICA algorithm. The ICs with intra-cluster similarity on average greater than 0.8 were selected and back-projected using the GICA back reconstruction algorithm. Z-score transformation was performed (Calhoun et al., 2001; Himberg et al., 2004). Meaningful ICs were selected *via* alignment with the typical resting-state network (RSN) template combined with manual check for two rounds following the standards provided by Allen et al. (2014) and Fiorenzato et al. (2019). Briefly, the selection standards were as follows: (1) ICs peaked majority in gray matter; (2) ICs were not predominantly located at blood vessels, ventricles, cerebrospinal fluid, white matter or regions outside the brain; (3) the main energy peaks distributed in the range of 0.01–0.1 Hz; (4) a wide dynamic range.

Eventually, 47 meaningful ICs were selected and sorted into cerebellum network (CBN) and 6 RSNs: basal ganglia network (BGN), default mode network (DMN), central executive network (CEN), sensorimotor network (SMN), auditory network (AN), and visual network (VN). Noise reduction was obtained to avoid the effects brought by physiological and scanner noises with the following post-processing procedures: de-trending of time courses (linear, quadratic and cubic); multiple regression with head motion parameters; removal of detected outliers; low-pass filtering with the high cut frequency of 0.15 Hz.

### Dynamic functional network connectivity assessment

The dFNC was analyzed with the functional network connectivity toolbox in GIFT using the sliding window and k-means clustering methods. With the sliding window method, the resting-state time series data were segmented into a 22-TR window with a size of 44 s (determined according to the study of Allen et al.), which was convoluted with the sigma 3-TR of the Gaussian function. The window was slid step-wise by 1 TR along the 200-TR length scan (400 s), resulting in 178 consecutive windows across the whole scanning process. The k-means clustering algorithm was applied on window functional connectivity matrices to assess the reoccurring functional connectivity patterns (states), which could be given as the frequency and structure of these states. The L1 distance (Manhattan distance), which has been proven to be an effective high-dimensional data measuring approach, was employed to evaluate the similarity between functional connectivity matrices. The reoccurring functional connectivity patterns were extracted by clustering analysis under each window and the optimal number of clusters was determined as 3. We obtained 3 major functional connectivity patterns from these data representative for intra- and inter-network dFNC states. Temporal properties of dFNC states were examined by assessing the following three variables: fractional windows, mean dwell time, and the number of transitions. Fractional window is the time spent in each state represented by percentage; mean dwell time is the time the participant spent in a certain state, which was an averaged value of the consecutive windows belonging to one state prior to changing to the other state; number of transitions is the number of transitioning between states, with a larger number representing lower stability of each state over time.

### Statistical analysis

Regarding statistical analysis for fractional windows, mean dwell time, and number of transitions, IBM SPSS 26.0 was applied. Friedman test and Bonferroni correction for multiple comparisons was adopted, with the threshold for statistical significance set at *P* < 0.05. The connection strength of the ICs between each state was extracted. The general characteristics of the connection status were obtained by comparing the means of the connection strength of all ICs in each sub-network, and the difference between states 1 and 3 was obtained by the Mann-Whitney *U*-test, with the threshold for statistical significance set at *P* < 0.05.

## Results

### Clinical manifestations

Following acute alcohol intake, most subjects showed alterations in mood, behavior, or skin of varying degrees, including dizziness (11/20), nausea (2/20), headache (1/20), unsteady gait (4/20), depression (1/20), excitement (5/20) and blush (11/20). There was no significant difference in BrAC between the 0.5 h group and the 1 h group (Supplementary Tables 3, 4 provide details of demographic and clinical data).

### Intrinsic network connectivity

**FIGURE 1:**
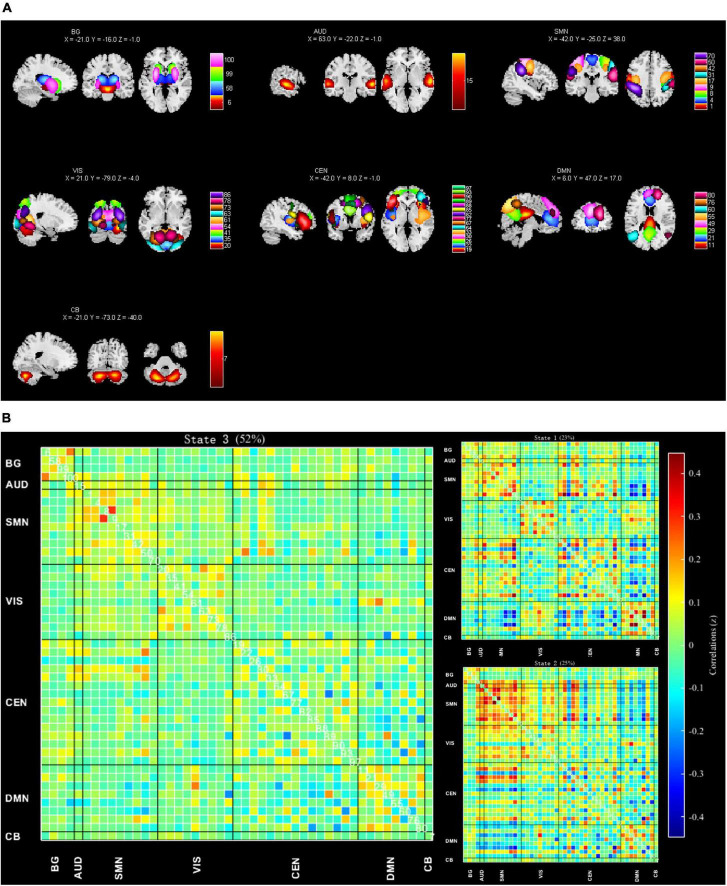
**(A)** Spatial maps of 47 identified meaningful independent components (ICs), sorted into seven functional networks: Basal ganglia (BG), auditory (AUD), sensorimotor (SMN), visual (VIS), central executive (CEN), default mode (DMN), and cerebellar networks (CB) (Supplementary Table 2 and Supplementary Figure 1 provides details of ICs). **(B)** Using the k-means clustering method, 3 FC states were extracted from dynamic FC data, and their centroids were displayed. Their occurrence rates are state 1 (23%), state 2 (25%), and state 3 (52%). Supplementary Material 2 presents the detailed connection strength of the three clusters.

Referring to the network distributions in the studies of Allen et al. (2014) and Fiorenzato et al. (2019), the current study, after performing spatial correlation analysis and visual inspection, assigned the 47 meaningful ICs into the seven following networks: BGN (*n* = 4), AN (*n* = 1), SMN (*n* = 9), VN (*n* = 9), CEN (*n* = 15), DMN (*n* = 8), and CBN (*n* = 1) (Figure 1A). Supplementary Table 2 and Supplementary Figure 1 presents the detailed component labels and peak coordinates of the 47 ICs.

### Dynamic functional network connectivity

Based on elbow criteria (Allen et al., 2014) and the optimal number of clusters as 3, we obtained 3 optimal dFNC states (Table 1 and Figure 1B). Supplementary Material 2 presents the detailed connection strength of the three clusters. In terms of occurrence rate, those of state 3 (52%) were higher than those of state 1 (23%) and state 2 (25%). State 1 is characterized by strong inter-network connections between BGN and SMN, BGN, and CEN, and DMN and VN. State 2 displayed overconnectivity between VN and SMN, VN, and CEN, and strong intra-network connections in SMN and VN. In contrast, state 3 is mainly characterized by relatively weak intra-network connections in SMN, VN, CEN, and DMN.

**TABLE 1:** The connection strength between and within the sub-network of the three clusters.

State	Sub-network	BG	AUD	SMN	VIS	CEN	DMN	CB
State 1	BG	0.0931 ± 0.0436	0.0333 ± 0.0564	0.0661 ± 0.0579	−0.0123 ± 0.0430	0.0382 ± 0.0865	−0.0427 ± 0.0719	0.0041 ± 0.0499
	AUD	0.0333 ± 0.0564	*NONE*	0.0351 ± 0.0918	0.0268 ± 0.0591	0.0265 ± 0.0786	0.0127 ± 0.0711	–0.0103
	SMN	0.0661 ± 0.0579	0.0351 ± 0.0918	0.1122 ± 0.0999	−0.0212 ± 0.0651	0.0476 ± 0.1309	−0.0837 ± 0.1114	−0.0316 ± 0.0456
	VIS	−0.0123 ± 0.0430	0.0268 ± 0.0591	−0.0212 ± 0.0651	0.0893 ± 0.1015	−0.0365 ± 0.0750	0.0356 ± 0.0825	0.0268 ± 0.0609
	CEN	0.0382 ± 0.0865	0.0265 ± 0.0786	0.0476 ± 0.1309	−0.0365 ± 0.0750	0.0343 ± 0.1363	−0.0677 ± 0.1269	−0.0153 ± 0.0597
	DMN	−0.0427 ± 0.0719	0.0127 ± 0.0711	−0.0837 ± 0.1114	0.0356 ± 0.0825	−0.0677 ± 0.1269	0.1293 ± 0.1379	−0.00519 ± 0.0506
	CB	0.0041 ± 0.0499	–0.0103	−0.0316 ± 0.0456	0.0268 ± 0.0609	−0.0153 ± 0.0597	−0.00519 ± 0.0506	*NONE*
State 2	BG	0.1118 ± 0.0604	0.0469 ± 0.1237	0.0387 ± 0.0877	0.0123 ± 0.0545	0.0085 ± 0.0873	0.0011 ± 0.0765	0.0303 ± 0.0539
	AUD	0.0469 ± 0.1237	*NONE*	0.2013 ± 0.0839	0.0802 ± 0.0964	0.0512 ± 0.1608	−0.0738 ± 0.1118	0.0024
	SMN	0.0387 ± 0.0877	0.2013 ± 0.0839	0.1911 ± 0.0906	0.0809 ± 0.0866	0.0230 ± 0.1372	−0.0764 ± 0.1164	−0.0100 ± 0.0224
	VIS	0.0123 ± 0.0545	0.0802 ± 0.0964	0.0809 ± 0.0866	0.0577 ± 0.0995	−0.0063 ± 0.0852	−0.0187 ± 0.1027	0.0307 ± 0.0397
	CEN	0.0085 ± 0.0873	0.0512 ± 0.1608	0.0230 ± 0.1372	−0.0063 ± 0.0852	0.0112 ± 0.1245	−0.0379 ± 0.1204	−0.0256 ± 0.0385
	DMN	0.0011 ± 0.0765	−0.0738 ± 0.1118	−0.0764 ± 0.1164	−0.0187 ± 0.1027	−0.0379 ± 0.1204	0.0695 ± 0.1285	−0.0050 ± 0.0358
	CB	0.0303 ± 0.0539	0.0024	−0.0100 ± 0.0224	0.0307 ± 0.0397	−0.0256 ± 0.0385	−0.0050 ± 0.0358	*NONE*
State 3	BG	0.1051 ± 0.0620	0.0133 ± 0.0638	0.0091 ± 0.0480***	−0.0202 ± 0.0243	0.0206 ± 0.0514	−0.0081 ± 0.0471**	0.0065 ± 0.0441
	AUD	0.0133 ± 0.0638	*NONE*	0.0643 ± 0.0457	0.0201 ± 0.0549	0.0271 ± 0.0674	−0.0244 ± 0.0530	0.0124
	SMN	0.0091 ± 0.0480***	0.0643 ± 0.0457	0.0760 ± 0.0620	0.0256 ± 0.0377***	0.0033 ± 0.0615***	−0.0251 ± 0.0521	−0.0016 ± 0.0261
	VIS	−0.0202 ± 0.0243	0.0201 ± 0.0549	0.0256 ± 0.0377***	0.0516 ± 0.0602	−0.0156 ± 0.0398**	−0.0034 ± 0.0602***	0.0076 ± 0.0471
	CEN	0.0206 ± 0.0514	0.0271 ± 0.0674	0.0033 ± 0.0615***	−0.0156 ± 0.0398**	0.0150 ± 0.0681	−0.0246 ± 0.0741***	−0.0153 ± 0.0330
	DMN	−0.0081 ± 0.0471**	−0.0244 ± 0.0530	−0.0251 ± 0.0521***	−0.0034 ± 0.0602***	−0.0246 ± 0.0741***	0.0497 ± 0.0868*	−0.0156 ± 0.0288
	CB	0.0065 ± 0.0441	0.0124	−0.0016 ± 0.0261	0.0076 ± 0.0471	−0.0153 ± 0.0330	−0.0156 ± 0.0288	*NONE*

The connection strength between and within sub-networks is presented in the form of (mean ± standard deviation). Each cell’s horizontal and vertical axesl correspond to the sub-networks involved in the connection strength. State 1 and state 3 are compared by Mann–Whitney U test, with the threshold for statistical significance set at P < 0.05, * indicates the significance of the difference (* for P < 0.05, ** for P < 0.01, *** for P < 0.001).

Compared with the control group, the fractional windows and mean dwell time in state 1 decreased in the 1 h group, and the fractional windows of 0.5 h group and 1 h group were increased in state 3 (Table 2). Table 2 presents the inter-group differences of dFNC temporal properties. Because not all subjects visited all states, the total number of subjects in each state varied (State 1, *N* = 44/60; State 2, *N* = 48/60; State 3, *N* = 57/60). Furthermore, when compared to the control group, the 0.5 h group and 1 h group had fewer subjects who visited state 1, while the control group had fewer subjects who visited state 3 (Table 3). No statistical significance in other indicators was observed in all between-group comparisons.

**TABLE 2:** dFNC temporal properties parameters in each group.

Parameter	Control group	0.5 h Group	1 h group
**Fraction (%)**			
State 1	0.192 (0.033–0.632)	0.108 (0.000–0.287)	0.036 (0.000–0.222)*
State 2	0.266 (0.042–0.371)	0.135 (0.060–0.394)	0.243 (0.000–0.509)
State 3	0.413 (0.292–0.606)	0.659 (0.383–0.780)*	0.527 (0.385–0.787)*
**Mean dwell time (s)**			
State 1	19.500 (5.500–32.375)	15.000 (0.000–21.563)	6.000 (0.000–17.250)*
State 2	21.250 (5.250–29.375)	18.250 (7.000–24.250)	18.000 (0.000–29.000)
State 3	23.625 (14.188–48.000	39.333 (24.417–59.583)	34.000 (27.500–64.250)
Transition	5.000 (2.250–7.000)	4.000 (2.250–6.000)	3.000 (2.000–6.000)

According to the type and distribution of the data, fractional windows, transition number, and mean dwell time are expressed as median (IQR). The brackets (after the median values) are stated (the 25–the75%). The groups after drinking and before drinking were compared, significant effects after correction for multiple comparisons (P < 0.05, Bonferroni corrected) are in bold (* for p < 0.05). There was no statistical difference between 0.5 h Group and 1 h Group.

**TABLE 3:** The number of subjects in each group visited different states.

Group	State 1	State 2	State 3
Control group	17 (85%)	17 (85%)	17 (85%)
0.5 h group	14 (70%)	17 (85%)	20 (100%)
1 h group	13 (65%)	14 (70%)	20 (100%)

The number of subjects from each group who visited each state. In parentheses, the percentage of subjects in the total is shown.

## Discussion

In this study, the sliding time window technique was used to analyze the changes in the resting-state functional brain network connectivity patterns that occurred before and after acute alcohol intake. We found that the dynamic properties of the functional brain network in volunteers changed after drinking, and the clinical manifestations may be related to the special network connection pattern in the corresponding state.

Using the K-means clustering algorithm, three relatively stable functional connectivity states were identified. State 1 showed the highest fractional windows and mean dwell time in the sober state before drinking, and the results were significantly different. Since state 1 is also the one with the largest difference in dynamic properties before and after drinking, it is plausible to hypothesize that state 1 is the main connection pattern in the wakeful state of the brain. In contrast, after drinking, the fractional windows and mean dwell time increased in state 3, and more subjects visited state 3 when they had drunk alcohol, suggesting that states 3 may be a post-drinking connection states. Indirect evidence for this can be seen in that state 3 showed the largest occurrence rate (52%), and that 66.7% of the imaging data in this study were taken after the volunteer had consumed alcohol. Below, we shall discuss this state’s characteristics and clinical significance and how it differs from state 1.

In terms of the characteristics of the connection pattern, different from state 1, state 3 displayed relatively weak intra-network connection in SMN, VN, CEN, and DMN. Moreover, compared with state 1, state 3 displayed augmented connectivity between SMN and VN, SMN and DMN, CEN and VN, CEN, and DMN, BGN, and DMN, and the results are statistically different. It suggested that the connection pattern of the functional brain network was reorganized after drinking, from the pattern dominated by intra-network connections to the pattern of enhanced inter-network connections. This transition was accomplished by adjusting the instantaneous frequency and dwell time of connection states of different patterns. Generally speaking, each sub-network has its own specific function, SMN manages somatosensory and motor abilities, VN controls vision, CEN mainly manages cognition and emotion, and DMN is associated with a variety of cognitive processes, including action, cognition, emotion, interoception, and perception (Buckner et al., 2008; Smith et al., 2009). The weakened connectivity within the network may reflect the dysfunction of the sub-network. It was consistent with volunteers’ motor, sensory, and cognitive impairment symptoms and mood changes after drinking. Combined with previous literature and the enhanced connectivity between sub-networks, we speculate that it may be related to alcohol-sensitive gamma aminobutyric acid (GABA) induction (Mihic and Harris, 1996; Fingelkurts et al., 2004; Lithari et al., 2012). Alcohol inhibits neuronal activity by interacting with GABA_A_ receptors on cell membranes, which in turn leads to increased functional connectivity between different brain regions. In conclusion, the connection pattern of state 3 may reflect the special mechanism of alcohol’s effect on functional brain networks, which induces drunkenness symptoms by inhibiting intra-network connections of sub-networks, while the enhancement of some inter-network connections is a compensatory change to maintain functional network normal operation. We only isolated one IC in each CBN and AN in this experiment, so we cannot see their intra-network connections. The effect of alcohol on CBN and AN is a well-established fact, and future attempts to segment them into smaller ICs may provide more informative trials (Sullivan et al., 1995; He et al., 2013; Choi et al., 2021).

On the other hand, compared with state 1, state 3 demonstrated weakened connectivity between BGN and CEN, and BGN and SMN after drinking, and there was a statistical difference in BGN and SMN. The BGN plays a role in motor function (Stocco et al., 2010). The basal ganglia can be thought of as a circuit whose function is to pick the action it wants and suppress the one it does not want. An impairment in the BG could trigger a reconstruction within the cortical-basal-cerebellar connection (Marczinski and Fillmore, 2003). Previous research (de Wit et al., 2000; Rzepecki-Smith et al., 2010) reported that basal ganglia activity is susceptible to acute alcohol intake and that acute alcohol exposure can impair the function of the basal ganglia, leading to the corresponding dysfunction. Therefore, we hypothesized that the weakened connectivity between the BGN and CEN, and BGN and SMN observed in state 3 results from an internal remodeling of the cortical-basal-cerebellar connectome and is associated with the motor and cognitive dysfunction observed in the volunteers. Rzepecki-Smith et al. (2010) demonstrated that alcohol causes a significant reduction in the connectivity between the frontotemporal basal ganglia and parts of the brain, which may lead to impairments in orderly cognitive and movement planning.

In conclusion, drinking can change the connection pattern of the functional brain network by adjusting the instantaneous frequency and dwell time of specific connectivity states. The change of connection pattern is manifested as the impairment of volunteers’ function (cognition, movement, sensation, self-control, etc.).

## Limitations

There are several limitations to this study. First, the sample size included in the experiment was small, and we believe that increasing the study’s sample size will enrich the fMRI data for better results. Considering the existence of multiple stages of drunkenness, setting different BrAC groupings is also considered helpful for the study. Secondly, obtaining volunteers’ symptoms is relatively subjective, and we think it is necessary to use professional scales to collect symptoms in the future. Finally, the optimal window size and the number of ICs are still debated, and its choice remains arbitrary. Regardless of these deficiencies, our findings still present the prospects of dFNC as a biomarker of alcohol-related behavioral changes.

## Conclusion

This experiment used the dFC method for the first time to investigate the connection mode of the resting-state functional brain network before and after acute alcohol intake. It was discovered that was a transient change in the connection patterns of functional brain networks following acute alcohol consumption, and there may be relatively independent characteristic functional network connection patterns, and the corresponding patterns characterize the clinical manifestations of volunteers. Dynamic functional connectivity helps reveal the changes in brain function under acute alcohol exposure, which may be a powerful tool for investigating the mechanism of alcohol-brain-related neural action.

## Data availability statement

The original contributions presented in this study are included in the article/Supplementary material, further inquiries can be directed to the corresponding author/s.

## Ethics statement

The studies involving human participants were reviewed and approved by the Ethics Committee of the Second Affiliated Hospital of Shantou University Medical College. The patients/participants provided their written informed consent to participate in this study.

## Author contributions

NL, HL, and WZ: conceptualization. GZ, HL, HZ, and WZ: data curation. HZ and WZ: investigation. HL and NL: methodology. NL, GZ, HZ, and WZ: resources. NL, GZ, and WZ: writing—original draft. All authors contributed to the article and approved the submitted version.

## Footnotes

1
http://icatb.sourceforge.net
