# Supplementary material for: Dynamic connectivity patterns of resting-state brain functional networks in healthy individuals after acute alcohol intake
Source: Front Neurosci. 2022 Sep 20;16:974778. doi: 10.3389/fnins.2022.974778 (PMC9531019; doi:10.3389/fnins.2022.974778)
Supplement: Supplementary file 1 [file Data_Sheet_1.docx]

**Supplementary Materials**

**Table S1. The head motion between groups**

|  | **Control Group** | **0.5h Group** | **1h Group** | **P-value** |
| --- | --- | --- | --- | --- |
| **FD** | 0.074(0.055,0.147) | 0.079(0.062,0.126) | 0.089(0.050,0.123) | 0.247 |

Continuous variables were expressed as median and interquartile ranges. Continuous parameters were checked for the normality of distribution using the Shapiro-Wilk test and compared using the Friedman test. A value of P<0.05 was considered statistically significant. Abbreviation: FD, frame-wise displacement.

**Table S2. Peak activations and coordinates of the ICN spatial maps.**

| **Number** | **ICNs** | **X** | **Y** | **Z** |
| --- | --- | --- | --- | --- |
|  | **Basal ganglia network （4）** | | | |
| 1 | Subthalamus/hypothalamus (6) | 2.5 | -17.5 | -14.5 |
| 2 | Caudate (58) | 20.5 | -15.5 | 12.5 |
| 3 | Putamen (99) | 20.5 | 15.5 | -0.5 |
| 4 | Putamen (100) | -26.5 | 2.5 | -5.5 |
|  | **Auditory domain (AUD)（1）** | | | |
| 5 | Superior temporal gyrus ([STG], 15) | 62.5 | -20.5 | -0.5 |
|  | **Sensorimotor domain (SM)（9）** | | | |
| 6 | Postcentral gyrus ([PoCG], 1) | -54.5 | -11.5 | 32.5 |
| 7 | Paracentral lobule ([ParaCL], 4) | -6.5 | -36.5 | 66.5 |
| 8 | Right postcentral gyrus ([R PoCG], 8) | 41.5 | -27.5 | 59.5 |
| 9 | Left postcentral gyrus ([L PoCG], 9) | -41.5 | -27.5 | 59.5 |
| 10 | Postcentral gyrus ([PoCG], 17) | 44.5 | -8.5 | 48.5 |
| 11 | Right postcentral gyrus ([R PoCG], 31) | 53.5 | -30.5 | 50.5 |
| 12 | Superior parietal lobule ([SPL], 42) | -20.5 | -54.5 | 63.5 |
| 13 | Postcentral gyrus ([PoCG], 50) | 57.5 | -27.5 | 24.5 |
| 14 | Left postcentral gyrus ([L PoCG], 70) | -44.5 | -36.5 | 38.5 |
|  | **Visual domain (VS)（8）** | | | |
| 15 | Right fusiform (20) | 32.5 | -77.5 | -9.5 |
| 16 | Cuneus (35) | 8.5 | -87.5 | -0.5 |
| 17 | Superior occipital gyrus ([SOG], 41) | -20.5 | -72.5 | 59.5 |
| 18 | Cuneus (54) | 11.5 | -84.5 | 20.5 |
| 19 | Calcarine gyrus ([CalcarineG], 61) | -17.5 | -62.5 | 9.5 |
| 20 | Middle occipital gyrus ([MOG], 63) | -29.5 | -92.5 | -6.5 |
| 21 | Lingual gyrus ([LingualG], 73) | -27.5 | -71.5 | 5.5 |
| 22 | Lingual gyrus ([LingualG], 78) | -15.5 | -54.5 | -12.5 |
| 23 | Superior occipital gyrus ([SOG], 86) | 33.5 | -74.5 | 17.5 |
|  | **Cognitive-control domain (CC)（15）** | | | |
| 24 | aInsula (19) | 32.5 | 27.5 | -0.5 |
| 25 | pInsula (22) | -41.5 | -5.5 | 11.5 |
| 26 | Middle frontal gyrus ([MiFG], 26) | -29.5 | 57.5 | 12.5 |
| 27 | Bi supplementary motor area (30) | 12.5 | -11.5 | 47.5 |
| 28 | pInsula (33) | 44.5 | -8.5 | 8.5 |
| 29 | Inferior frontal gyrus ([IFG], 64) | 36.5 | 42.5 | -3.5 |
| 30 | Right inferior parietal lobule([R IPL], 67) | 42.5 | -69.5 | 44.5 |
| 31 | Middle frontal gyrus ([MiFG], 77) | -23.5 | 39.5 | 21.5 |
| 32 | Right inferior frontal gyrus ([R IFG], 82) | 51.5 | 23.5 | 23.5 |
| 33 | Inferior frontal gyrus ([IFG], 85) | 51.5 | 5.5 | 18.5 |
| 34 | Superior frontal gyrus ([SFG], 88) | 17.5 | 2.5 | 54.5 |
| 35 | Supplementary motor area([SMA], 89) | -6.5 | 0.5 | 66.5 |
| 36 | Left inferior frontal gyrus ([L IFG], 90) | -44.5 | 29.5 | 5.5 |
| 37 | Middle cingulate cortex ([MCC], 93) | -3.5 | 6.5 | 33.5 |
| 38 | Middle cingulate cortex ([MCC], 97) | 8.5 | 26.5 | 44.5 |
|  | **Default-mode domain (DM)（8）** | | | |
| 39 | Posterior cingulate cortex ([PCC], 11) | -2.5 | -26.5 | 23.5 |
| 40 | Anterior cingulate cortex ([ACC], 21) | -0.5 | 41.5 | 11.5 |
| 41 | Posterior cingulate cortex ([PCC], 29) | -2.5 | -62.5 | 26.5 |
| 42 | Bi Medial superior frontal ( 49) | -5.5 | 50.5 | 29.5 |
| 43 | Precuneus+Cuneus (55) | 5.5 | -69.5 | 56.5 |
| 44 | Left Angular gyrus ([LAG], 60) | -47.5 | -62.5 | 27.5 |
| 45 | Precuneus (76) | -14.5 | -53.5 | 51.5 |
| 46 | Right Angular gyrus+Right superior frontal gyrus (80) | 21.5 | 48.5 | 23.5 |
|  | **Cerebellar domain (CB)（1）** | | | |
| 47 | Cerebellum ([CB], 7) | -21.5 | -71.5 | -38.5 |

**Table S3. Demographic and Clinical Data**

| **Subject** | **[Gender](C:/Users/Administrator/AppData/Local/youdao/dict/Application/8.10.3.0/resultui/html/index.html#/javascript:;)** | **Age** | **Weight**  **(kg)** | **Alcohol consumption (ml)** | **BrAC（mg/L）** | | **Symptoms** |
| --- | --- | --- | --- | --- | --- | --- | --- |
|  |  |  |  |  | **0.5h** | **1h** |  |
| Sub01 | Female | 25 | 57.5 | 56 | 0.40 | 0.25 | Dizziness |
| Sub02 | Male | 24 | 57 | 56 | 0.65 | 0.50 | Excitement, increased speech |
| Sub03 | Female | 25 | 53 | 52 | 0.50 | 0.55 | Excitement, increased speech |
| Sub04 | Male | 31 | 78 | 77 | 0.40 | 0.20 | Dizziness, Depression, Blushing, Walking unsteadily |
| Sub05 | Male | 23 | 55 | 54 | 0.25 | 0.40 | Dizziness |
| Sub06 | Male | 27 | 105 | 103 | 0.75 | 0.50 | Dizziness, Blushing, Walking unsteadily |
| Sub07 | Male | 23 | 65 | 64 | 0.35 | 0.50 | Dizziness, Blushing |
| Sub08 | Male | 26 | 75 | 74 | 0.50 | 0.35 | Dizziness, Headache, Blushing, Nausea |
| Sub09 | Female | 23 | 60 | 59 | 0.40 | 0.50 | None |
| Sub10 | Male | 26 | 55 | 54 | 0.30 | 0.50 | Excitement, increased speech |
| Sub11 | Female | 23 | 45 | 44 | 0.25 | 0.35 | None |
| Sub12 | Female | 26 | 57 | 56 | 0.40 | 0.50 | Dizziness, Blushing, Walking unsteadily |
| Sub13 | Female | 25 | 41 | 40 | 0.25 | 0.45 | None |
| Sub14 | Female | 23 | 43 | 42 | 0.45 | 0.30 | Dizziness, Blushing |
| Sub15 | Male | 25 | 74 | 73 | 0.30 | 0.15 | Blushing |
| Sub16 | Male | 23 | 85 | 83 | 0.20 | 0.50 | Excitement, Blushing, increased speech |
| Sub17 | Male | 23 | 83 | 81 | 0.25 | 0.40 | Excitement, Blushing |
| Sub18 | Male | 30 | 65 | 64 | 0.50 | 0.40 | Dizziness, Nausea, Blushing, Walking unsteadily |
| Sub19 | Female | 26 | 50 | 49 | 0.35 | 0.15 | Dizziness |
| Sub20 | Female | 25 | 53 | 52 | 0.30 | 0.50 | Dizziness, Blushing |

**Table S4. The BrAC between groups**

|  | **0.5h Group** | **1h Group** | **P-value** |
| --- | --- | --- | --- |
| **BrAC** | 0.425(0.313,0.500) | 0.375(0.263,0.488) | 0.9100 |

Continuous variables were expressed as median and interquartile ranges. Continuous parameters were checked for the normality of distribution using the Shapiro-Wilk test and compared using Wilcoxon and Mann-Whitney U tests. A value of P<0.05 was considered statistically significant. Abbreviation: BrAC, breath alcohol concentration.

**FIGURE S1**

**Basal ganglia network**


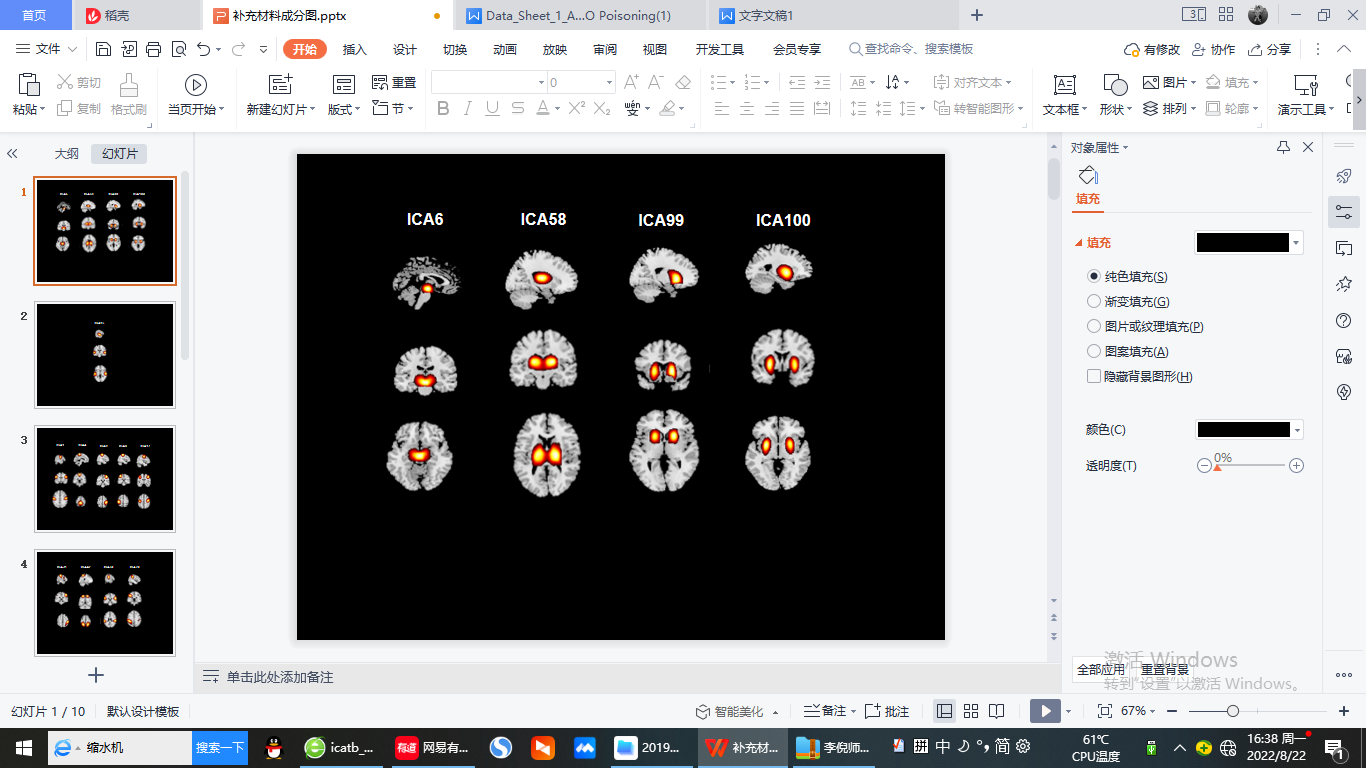


**Auditory network**


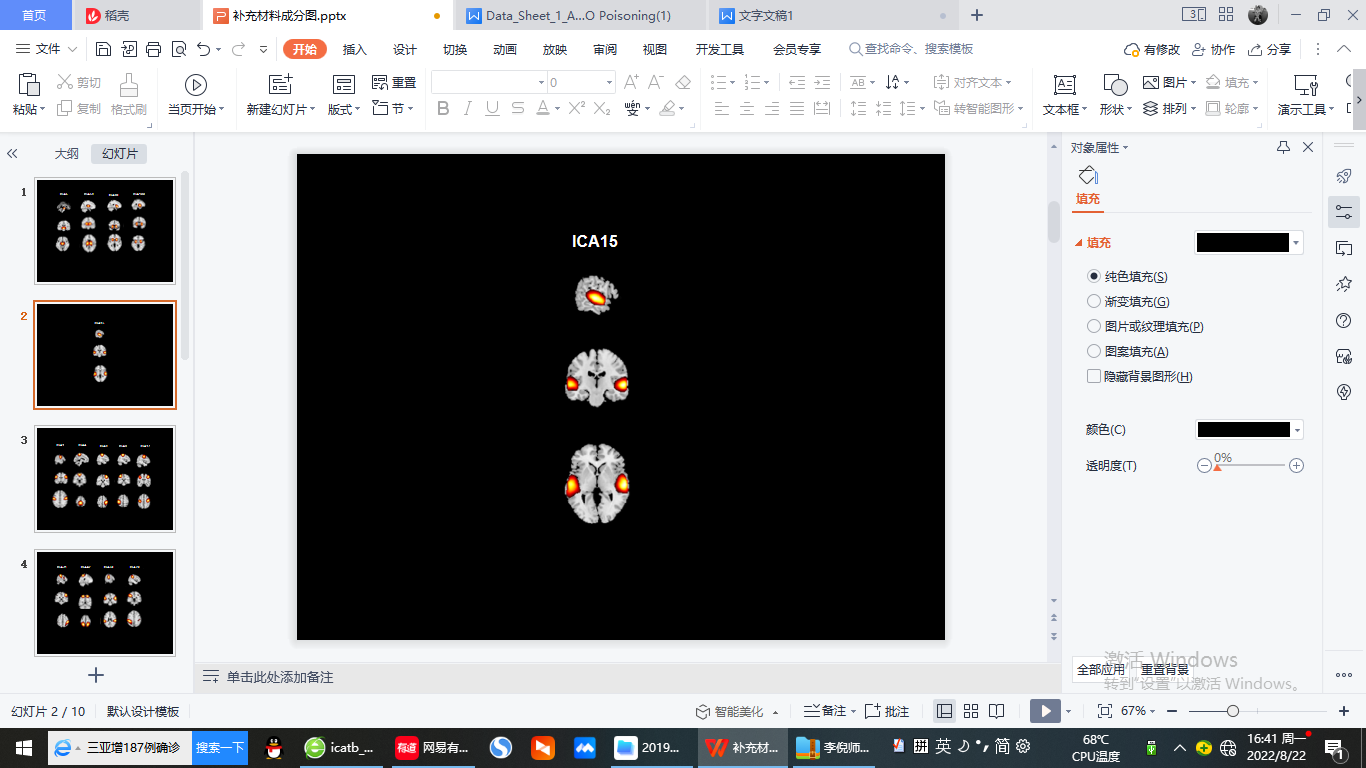


**Sensorimotor network**


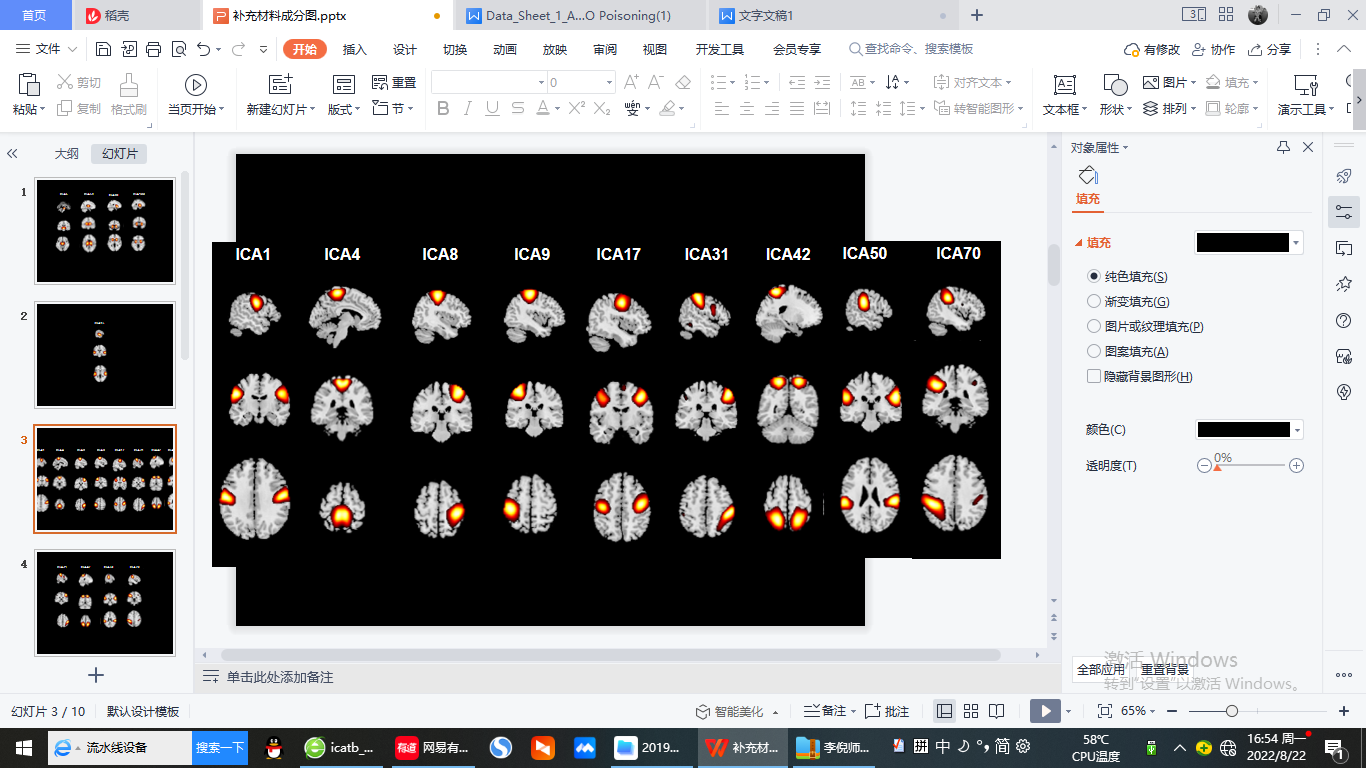


**Visual network**


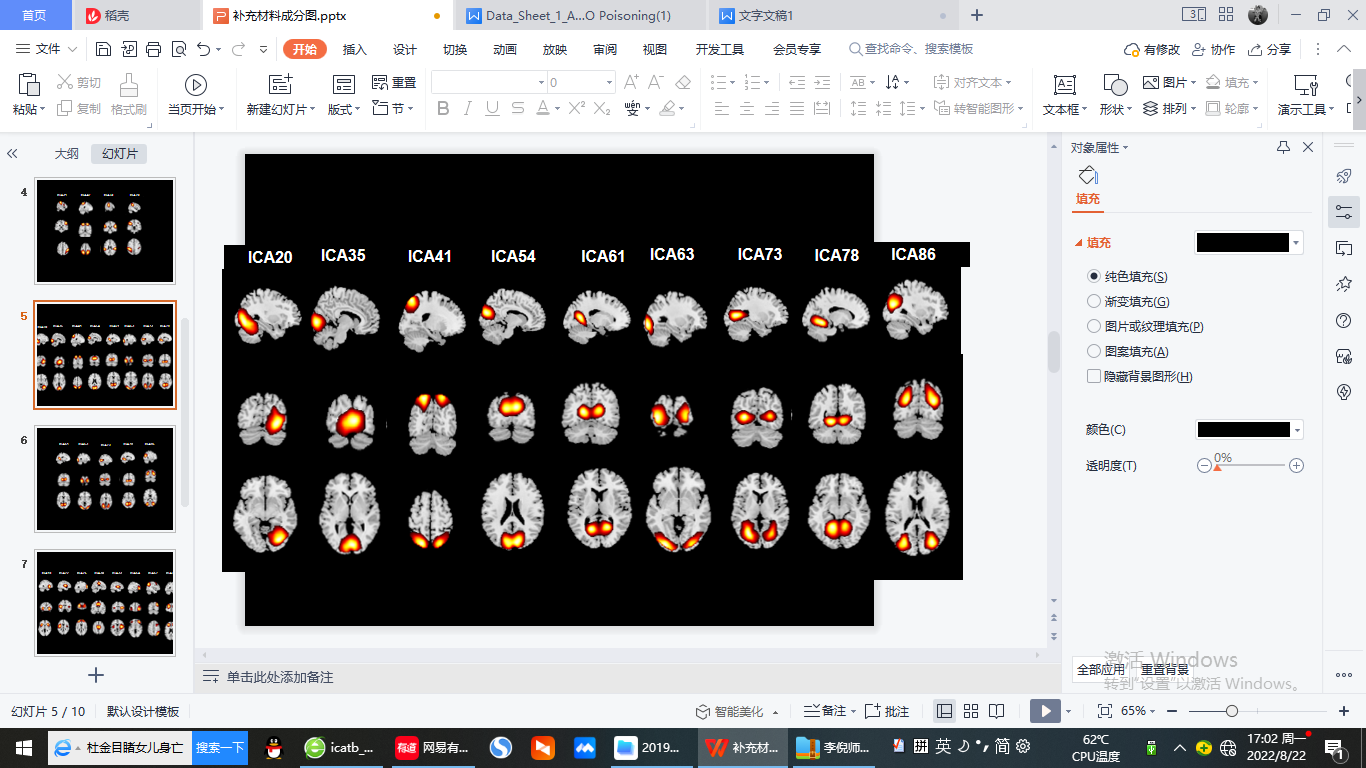


**Cognitive executive network**


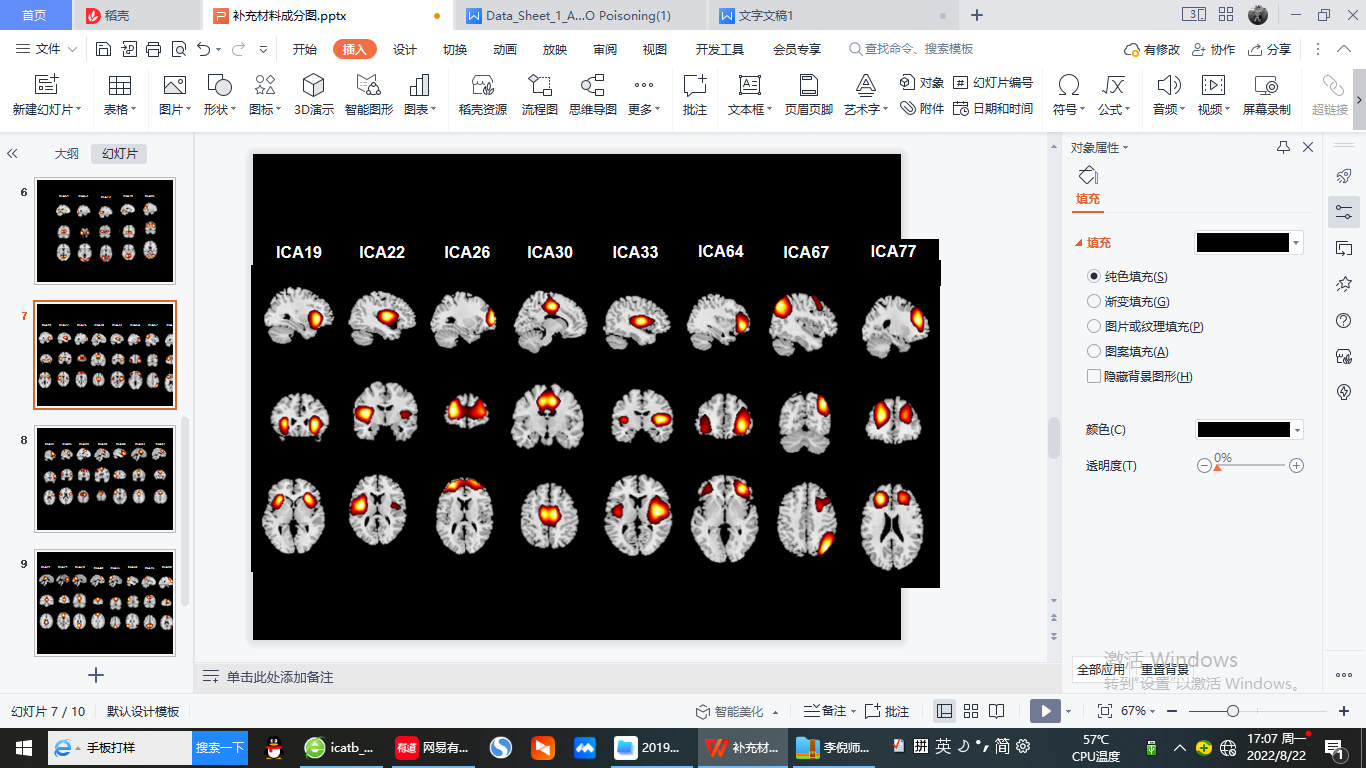


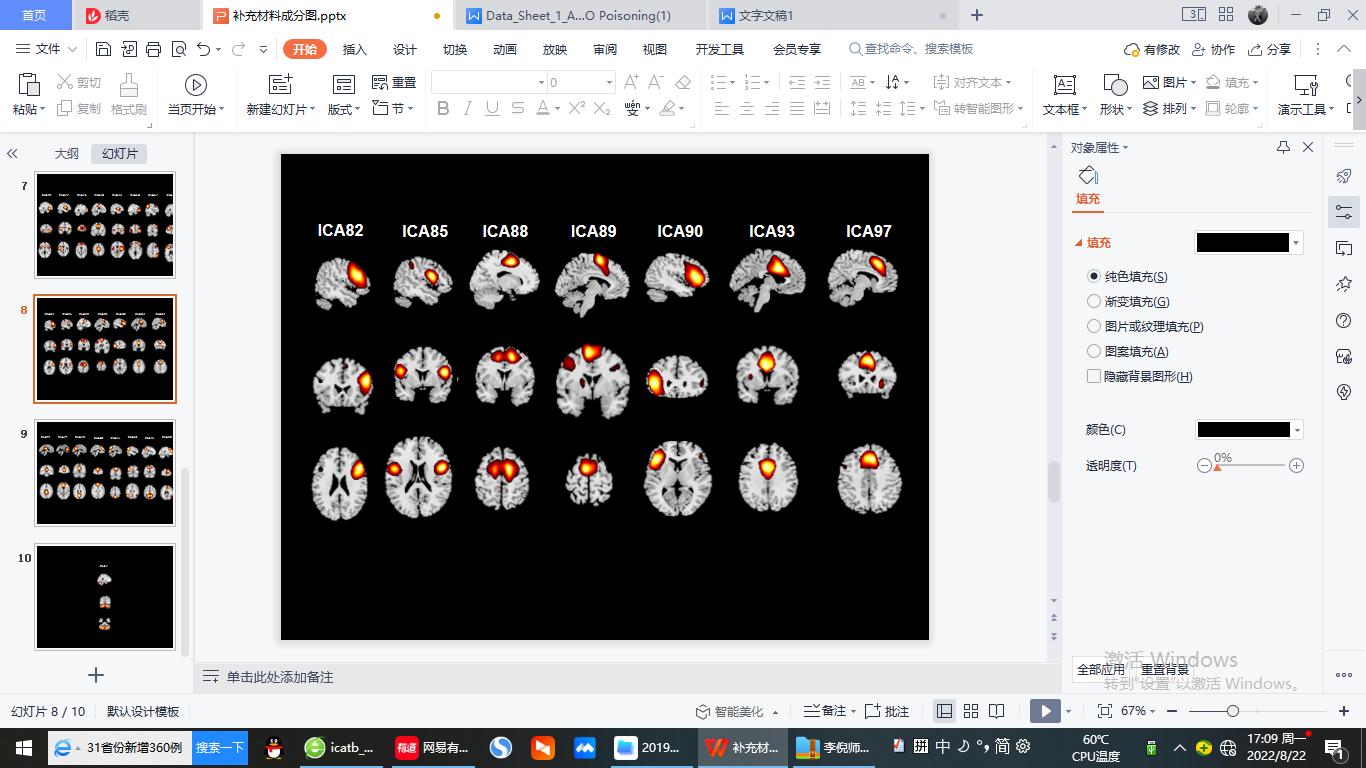


**Default mode network**


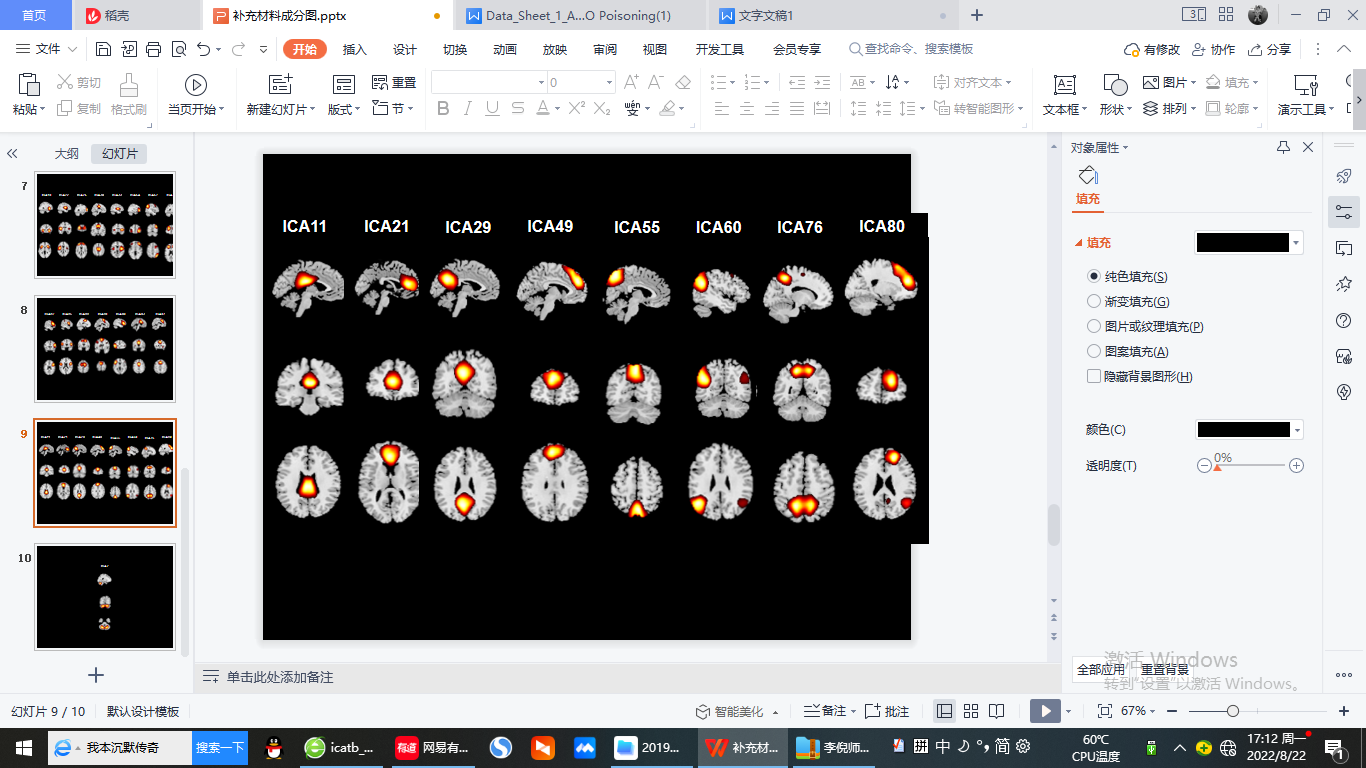


**Cerebellar network**


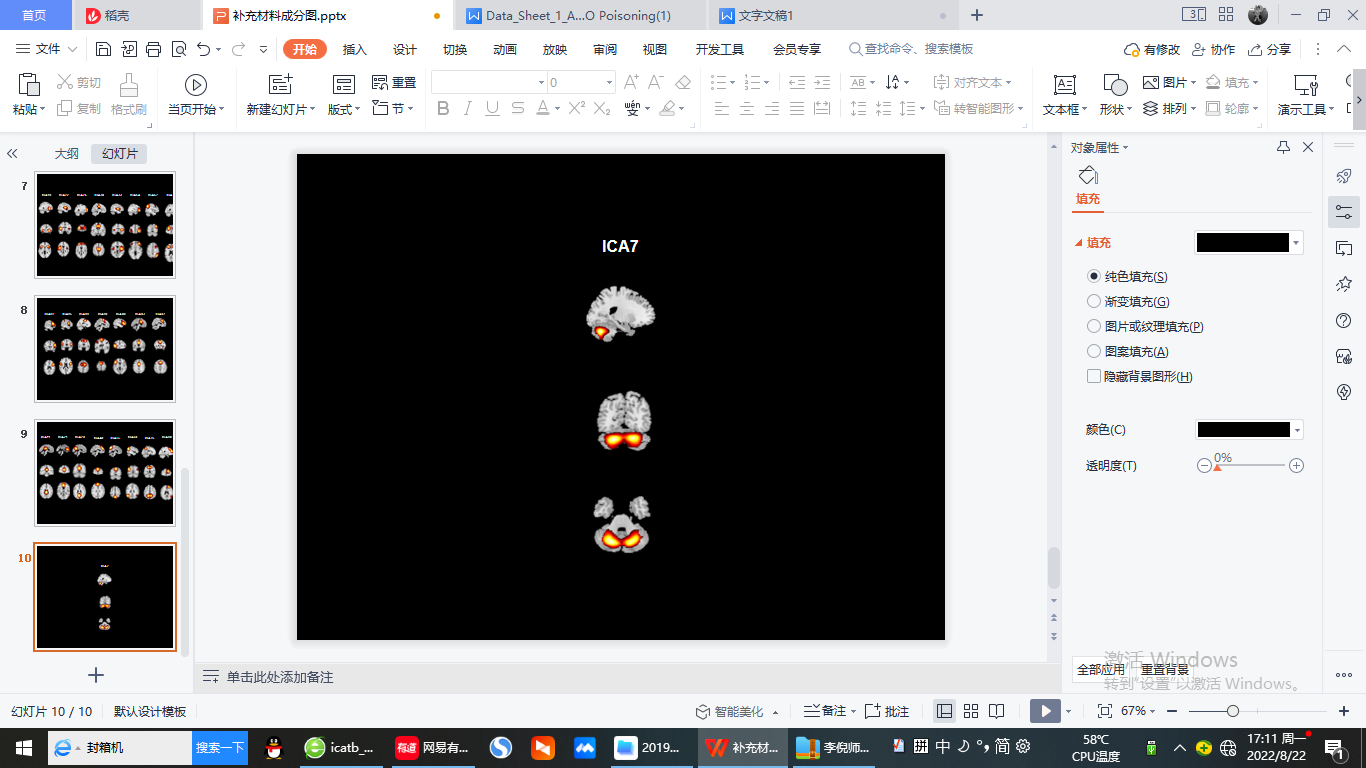


**FIGURE 1** | Spatial maps of 47 identified meaningful independent components (ICs), sorted into seven functional networks: Basal ganglia (BG), auditory (AUD), sensorimotor (SMN), visual (VIS), central executive (CEN), default mode (DMN) and cerebellar networks (CB).
